# Assessment of Physicochemical and Rheological Properties of Xylo-Oligosaccharides and Glucose-Enriched Doughs Fermented with BB-12

**DOI:** 10.3390/biology11040553

**Published:** 2022-04-02

**Authors:** Gabriela Precup, Bernadette-Emőke Teleky, Floricuța Ranga, Dan Cristian Vodnar

**Affiliations:** 1 Faculty of Food Science and Technology, University of Agricultural Sciences and Veterinary Medicine, Calea Mănăstur 3-5, 400372 Cluj-Napoca, Romania; gabriela.precup@usamvcluj.ro (G.P.); floricutza_ro@yahoo.com (F.R.); 2 Institute of Life Sciences, University of Agricultural Sciences and Veterinary Medicine, Calea Mănăstur 3-5, 400372 Cluj-Napoca, Romania

**Keywords:** xylo-oligosaccharides, sourdough, rheology, wheat flour, *Bifidobacterium bifido*

## Abstract

**Simple Summary:**

Xylo-oligosaccharides (XOS) are considered indigestible fibers that could support the growth of potentially beneficial gut microbes, thus classified as “prebiotics”. Prebiotics are “a substrate that is selectively utilized by host microorganisms conferring a health benefit” as defined by the International Scientific Association for Probiotics and Prebiotics. The current work aimed to study the effect of XOS and glucose addition on wheat flour sourdough fermented with *Bifidobacterium animalis* subsp. *lactis* (BB-12) strain in terms of organic acid production and on the rheological properties of the doughs. The effect of XOS addition increased the production of organic acids, and positively influenced the rheological properties of the dough. Additionally, after frozen storage, there were no significant viscoelastic changes in the dough structure, which indicates that xylo-oligosaccharides improved the water retention capability of the dough. Through fermentation carbohydrates like, glucose, xylose, maltose, and XOS were consumed, and a high quantity of lactic and acetic acid were produced, organic acids with roles in the flavor generation and sensorial properties of the final product. This study showed the potential use of XOS as food ingredient in sourdoughs for bakery products manufacturing with improved quality and rheological properties.

**Abstract:**

Xylo-oligosaccharides (XOS) are considered non-digestible fibers produced mainly from agricultural biomass and are classified as “emerging prebiotic” compounds. Since XOS were shown to promote the growth of bifidobacteria in the gut with potential effects on one’s health, scientists used them as food ingredients. For example, the addition of XOS in bakery products could improve their physicochemical characteristics. The current work aimed to investigate the effect of XOS and glucose addition on wheat flour sourdough fermented with *Bifidobacterium animalis* subsp. *lactis* (BB-12) strain in terms of organic acid production. The effect on viscoelastic changes during frozen storage and after the thawing process was also studied. The results showed that the viability of BB-12 increased slightly with the increase in XOS and glucose concentrations, which determined dough acidification due to accumulation of organic acids, that positively influenced the dough’s rheological properties such as a higher elasticity before and after frozen storage. With 10% XOS-addition, the acetic acid quantity reached 0.87 ± 0.03 mg/L, and the highest lactic acid concentration was found in the 10% XOS-enriched doughs, the glucose-enriched doughs and in the control sample (100% wheat dough). The quantity of glucose, maltose, XOS, and xylose decreased until the end of fermentation.

## 1. Introduction

Emerging evidence in recent decades from clinical and animal research has shown the impact of the gut microbiota on host health along the microbiota–gut–brain axis [1,2,3]. Dysbiosis in the intestinal microbial communities was associated with developing non-communicable diseases, such as type 1 diabetes, obesity, inflammatory bowel disease, irritable bowel syndrome, colorectal cancer, rheumatoid arthritis, allergies, autism, and major depressive disorders [4,5]. A promising approach through dietary and therapeutic interventions targeting the consequences of intestinal imbalances could be given by using pre-, pro-, and synbiotics to regulate the gut microbiota by producing short-chain fatty acids, lowering the intestinal pH, and supporting mineral absorption [6,7]. In addition, given the outbreak of the coronavirus pandemic (COVID-19) [8] and its psychological burden, the administration of the aforementioned compounds were shown to possibly have supportive roles in enhancing the immune system [9,10], as well as putative beneficial effects in other metabolic, cardiovascular, psychiatric, and neurological disorders [11].

Prebiotics were recently defined by the ISAPP (International Scientific Association for Probiotics and Prebiotics) as “a substrate that is selectively utilized by host microorganisms conferring a health benefit” [12]. Prebiotics are mainly represented by nutrients such as oligosaccharides (inulin and fructo-, galacto-, isomalto-, mannano-, raffinose, and xylo-oligosaccharides) [13]. Xylo-oligosaccharides (XOS) are considered non-digestible fibers generally produced from xylan-rich biomasses that can be found naturally in fruits, vegetables, milk, honey, or even bamboo shoots [14]. Since most foods contain only trace levels of these nutrients, researchers exploited the use of agricultural leftovers via enzymatic, thermal, or chemical processes for XOS production [13,15]. Recently, XOS were added as food ingredients in various food categories (cookies, dairy products, beverages, fruit juices, and chewing gums) [16,17] and were shown to remain unaltered during exposure to heat and acidic foods, with a sweetness of 40–50% of sucrose and a caloric amount of 4.0 kcal/g [18]. In Japan and China, XOS are available as food ingredients and food supplements, respectively. In the EU, XOS derived from corn cobs and produced via enzyme-catalyzed hydrolysis were authorized by the European Commission in 2018 to be placed on the market in various food categories as novel food (NF) ingredients after their safety for human consumption was assessed by the European Food Safety Authority (EFSA) [19,20]. However, no assessment of the efficacy of the NF concerning any claimed benefit was performed. Nevertheless, in vitro and in vivo studies linked XOS with health outcomes related to the gut microbiota [21,22]. Specifically, dietary interventions with XOS showed an increase in the abundance of colonic lactic acid bacteria (*Bifidobacterium* spp.) and *Faecalibacterium prausnitzii*, which were demonstrated to have low abundance in obese individuals and were linked to health outcomes such as weight decrease, reduced risk of infections for infants, or improving gastrointestinal infections [23,24,25]. These bacteria are considered “probiotics”, referring to “live microorganisms that, when administered in adequate amounts, confer a health benefit on the host”, in accordance with the World Health Organization and the Food and Agricultural Organization of the United Nations [26,27]. According to ISAPP recommendation, until this moment just some specific strains have been characterized, thus various strains belonging to the same species can exert distinct health effects [28].

The techno-functional properties of “probiotic” microorganisms used for food applications were also investigated in several papers [29,30,31,32]. In particular, *Bifidobacterium animalis subsp. lactis* (BB-12^®^), a rod-shaped, catalase-negative bacterium isolated since 1983, was easily cultivated and demonstrated good tolerance to oxygen and acid and excellent stability in fermentation processes [33]. In addition, the mechanism of action was indicated as using the bifidus pathway, specifically through the use of fructose-6-phosphate phosphoketolase, which enables the bacteria to generate from carbohydrates’ additional ATP [34]. Fermentation studies with BB-12 on XOS used as carbohydrate source showed potential prebiotic activity by increasing the microbial production of short-chain fatty acids (e.g., acetate, butyrate), which supported the immune system [35,36]. Generally recognized as safe (GRAS) in the Unites States of America and with granted qualified presumption of safety (QPS) status in the European Union, BB-12 is a microorganism that can be included in several food applications, such as in fermented dairy products, as a sugar substitute, in cereal, and in nutritional drinks [37]. Although it was primarily classified as anaerobic, several studies proved that it is tolerant to heat, oxygen, and acidic pH [38]. The inclusion of this probiotic microorganism in bakery products, although with minimal survival, can confer several positive characteristics to the final product. Bifidobacteria are able to generate vitamins, and they can ferment carbohydrates (i.e., glucose, XOS, FOS).

Limited information is available about the effect of XOS on the quality of food products, such as bakery foodstuffs. Previous studies suggested that XOSs could have a flavor-enhancer role and be considered as an alternative to increasing the dietary fiber content [39]. A recent study analyzed the effect of XOS supplementation on cookie doughs, which made them crispier, provided enhanced caramel flavor, and amplified the baking process [16]. Frozen storage, which implies three stages—pre-cooling, phase transition, and tempering—is frequently employed in dough conservation; however, it also presents some drawbacks. The second stage of freezing, namely phase transition, implies the transition of water to ice through crystallization, which is critical in process efficiency [40]. During the generation of ice crystals, bakery products experience physical detriment, followed by inadequate loaf volume, diminished gas-holding capacity, and a substantial weakening of dough physicochemical characteristics [41]. Thus, the quality and nutritional enrichment of bakery products are highly researched, with specific purposes regarding improved shelf life and quality preservation through frozen storage and during the thawing process [42,43].

Therefore, in this study, we aimed to investigate the rheological and physicochemical effects of supplementation of wheat flour (WF) doughs with XOS obtained by chemical and enzymatic synthesis from wheat straw [14] by comparing them with glucose-enriched doughs. Moreover, the effects of fermentation with BB-12 under aerobic conditions on the enriched doughs were assessed after fermentation, frozen storage, and thawing, with an overall objective of improving the dough’s physicochemical properties.

## 2. Materials and Methods

### 2.1. Materials and Formulations

Wheat flour (WF) (*Triticum aestivum* L.) was obtained from commerce (Băneasa, type 000) with 15.3% moisture, 11.2% protein, 1.3% dietary fiber, 0.48% ash content, and without any additives. Several formulations of wheat flour and XOS/glucose were made as follows: 100% WF as control; WF with 1, 2, 5, and 10% XOS addition; and, as comparison, WF with 1, 2, 5, and 10% glucose addition. The concentrations of XOS and glucose were based on similar studies, although with different substrates and glucose [44,45,46].

### 2.2. Microorganisms and Culture Environment

As microorganism, *Bifidobacterium bifido* subsp. *lactis* (BB-12) from Chr. Hansen Inc. (Milwaukee, WI, USA) was used during the study, which has proven tolerance to heat and oxygen [47]. BB-12 was received from the University of Agricultural Sciences and Veterinary Medicine Cluj-Napoca. As fermentation medium, De Man, Rogosa, and Sharpe broth (MRS broth: glucose 20 g/L; meat extract 10 g/L; casein peptone-tryptic digest 10 g/L; yeast extract 5 g/L; Na-acetate 5 g/L; K_2_HPO_4_ 2 g/L; (NH_4_)_3_ citrate 2 g/L; Tween 80 1 g/L; MgSO_4_ × 7 H_2_O 0.2 g/L; MnSO_4_ × H_2_O 2 g/L; distilled water 1 L) was utilized [48]. The reactivation of BB-12 before the experimental application was 1 mL of microorganism in 9 mL of MRS broth. The vial was incubated at 30 °C for a period of 18–24 h, and afterward, it was propagated (10 mL) in 90 mL of fresh media and re-incubated for 18–24 h. A BB-12 concentration of log_10_ 10^8^ colony-forming units per milliliter (CFU/mL) was established with a NanoDrop 1000 spectrophotometer (NanoDrop Technologies, Wilmington, DE, USA) within 0.009 and 0.011 values measured at an optical density of 600 nm (OD600) [29]. Additionally, with the help of a microscope (Nikon, Tokyo, Japan), the probability of contamination was also eliminated.

### 2.3. Sourdough Formulation and Fermentation

Sourdough preparation was made according to previous studies [45,49], with every experiment repeated three times in special Duran bottles. The added water volume (water and the starter culture) was identical to the quantity of flour, with a final yield of 300 g of sourdough. First, the flour was sterilized. The necessary quantity of XOS or glucose (1, 2, 5, or 10%) was introduced in the distilled water, then homogenized and sterilized in an autoclave for 20 min at 121 °C (Autoclave 4002136, J.P. Selecta, Barcelona, Spain). Under the sterile bench, the WF and water were thoroughly mixed with the inoculated 30 mL of BB-12 starter culture for a minimum of 1 min and fermented for 48 h. From every batch, samples were prelevated at 0, 18, 24, and 48 h for pH (5 g), cell viability (1 g), HPLC (5 g), and rheology (5 g), after which they were incubated at 30 °C. Sample prelevation was performed with a sterile spatula and weighing boats (Whatman^®^).

### 2.4. pH and Cell Viability

pH measurement, thorough the in vitro experiments, was determined with the help of a digital pH meter (InoLab 7110, Wellheim, Germany) at the same temperature (25 °C) [50,51]. A quantity of 5 g of sourdough prelevated from each experiment was diluted in 45 mL of distilled water and homogenized on a magnetic stirrer (IKA^®^, RCT basic, Staufen, Germany).

To determine cell viability, 1 g of sample was prelevated and diluted in 9 mL of sterile saline solution (0.8% NaCl), after which a 5-fold or 7-fold dilution was made. In a Petri dish with the pour plate method, 1 mL of diluted inoculum and a quantity of approx. 15 mL lukewarm MRS agar was poured, homogenized, and left until it became solid. The plates (triplicate) were incubated at 30 °C for 48 h and counted afterwards.

### 2.5. Secondary Metabolite Analysis by HPLC-RID

Following fermentation, the samples (triplicates) were prepared for analysis. First, 1 g of sourdough was diluted in 2 mL of distilled water, vortexed for 30 s, sonicated for 15 min, and centrifuged (10 min at 8000 rpm at 4 °C). Next, the supernatant was filtered (0.45 µm Millipore membrane filter), and a sample quantity of 20 µL was injected in the column of a high-performance liquid chromatograph (HPLC-Agilent 1200 series, Santa Clara, CA, USA) over a flow rate of 0.5 mL/min. Then, the identification was carried out at 280 and 340 nm [52]. The utilized HPLC was equipped with a manual injector associated with a refractive index detector (RID) (Agilent Technologies, Santa Clara, CA, USA), a solvent degasser, and a quaternary pump. The compounds were severed on a Polaris Hi–Plex H column (300 × 7.7 mm) (Agilent Technologies, Santa Clara, CA, USA), utilizing the mobile phase (5 mM H_2_SO_4_) with a 0.6 mL/min flow rate, 80 °C column temperature, and 35 °C RID temperature. Compound extraction was performed for 25 min. Data acquisition and results interpretation were performed utilizing OpenLab software: ChemStation (Agilent Technologies, Santa Clara, CA, USA). Compound detection was accomplished by comparing the retention times between the standard compounds and the analyzed samples. The measured compounds were maltose, glucose, XOS, fructose, lactic, and acetic acid.

### 2.6. Rheological Measurements

After prelevation, the samples (triplicates) were analyzed in a fresh state and stored at −20 °C (frozen storage) for one month. At the time point of 4 weeks, the samples were reanalyzed after thawing at room temperature. Measurements were performed with an Anton Paar MCR 72 rheometer (Anton Paar, Graz, Austria) equipped with a temperature-regulated Peltier plate-plate system (P-PTD 200/Air) with a 50 mm width smooth parallel-plate geometry (PP-50-67300). A quantity of around 3 g of dough was supplied on the lower plate, and after that, the upper plate was descended with a distance between the plates of 1 mm. Dough surplus was eliminated, samples were left to rest for 10 min to enable dough relaxation, and silicone oil was supplemented on the outside of the upper plate geometry to prevent sample drying during the experiment [44,53].

The dynamic rheological features of the samples were assessed through oscillatory frequency sweep test at an angular frequency (ω) from 0.628 to 628 rad/s, which was based on the previously determined linear viscoelastic zone determined at 6.28 rad/s stress sweep assay. After the dynamic rheological measurement, the loss factor (damping factor) was also determined from the ratio of loss (G”) and storage (G’) modulus; otherwise, *δ* represents the phase angle within stress and strain (*σ*, *γ*), shown as:

tan*δ* = G”/G’;

which characterizes the relationship of the two parts of the viscoelastic demeanor [54].

### 2.7. Statistical Analysis

Each experiment and measurement was performed in triplicate, with the results reported as mean ± SD (standard deviation). Statistical evaluation was carried out through the use of Graph Prism Version 8.0.1. (GraphPad Software Inc., San Diego, CA, USA), along with a one-way ANOVA test (Tukey multiple comparisons tests) [55]. Statistically significant differences of means were considered at a level of *p* < 0.05.

## 3. Results and Discussions

### 3.1. pH and Cell Viability

**Figure 1:**
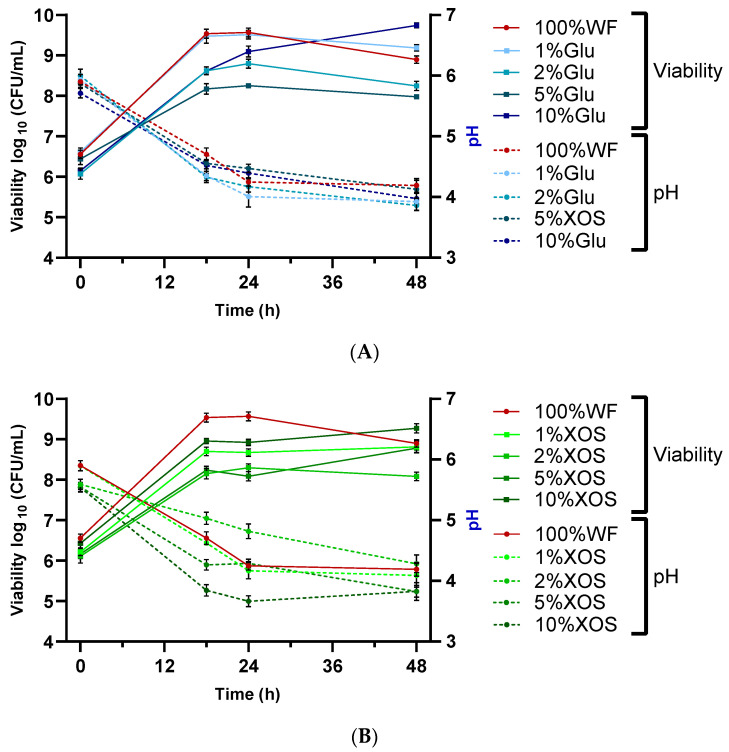
Cell viability (continuous line) and pH (dotted line) of dough fermented with BB-12. Three fermentation varieties with 100% WF were made: (**A**) with 1, 2, 5, and 10% glucose of addition and (**B**) with 1, 2, 5, and 10% of XOS addition. The viable cell counts of BB-12 and pH are presented as mean values ± SD, log_10_ CFU/mL, n = 3.

Although humans cannot digest nutrients such as XOS (thus providing no nourishment), they exert an important outcome and support the multiplication of “friendly” over “unfriendly” intestinal flora [56]. Furthermore, the utilization of XOS enhances the amount of intestinal *Bifidobacterium* in a daily portion as low as 1.4 g, compared to the mostly known fructo-oligosaccharides, which require a daily portion of more than 10 g per day [57]. To stimulate fermentation 1, 2, 5, and 10% of XOS or glucose was added to WF, and 100% WF was used as control through dough fermentation with BB-12 (Figure 1). The concentration was based on previous studies, where it was determined that supplementation with more than 10% high-fiber content negatively impacts the rheological properties of dough, the gluten network, and the final product [46].

In every dough composition, the growth of BB-12 was enhanced from a mean value of 6.10–6.60 log_10_ CFU/mL to 8.80–9.70 log_10_ CFU/mL. The highest value was observed in the batch with 10% glucose (9.74 ± 0.06), followed by 10% XOS (9.27 ± 0.11). The viability in the batch with 100% WF had an intermediate value of 8.90 ± 0.09, suggesting that the addition of glucose or XOS increased cell viability. During the fermentation period of 48 h, the viability increased, and the pH decreased. However, after 24 h, no significant viability increase could be observed, and the values dropped or remained constant, suggesting the end of fermentation. These results are in accordance with a similar result, where BB-12 grew well on glucose, oligofructose, and lactose but did not grow on fructose alone. The final cell count was above 1.0 × 10^9^ CFU ml^−1^, and in this case, BB-12 also produced succinic acid, in addition to lactic and acetic acid [58].

As indicated previously, with the increase in the viable cell count, the pH gradually decreased from pH 5.5–6.0 to values within the range of 3.8–4.3. This pH drop could be related to the generation of lactic acid (or in the case of XOS, addition of acetic acid) by BB-12. With the ongoing metabolic activity, the pH diminished until it obtained a critical level that could also inhibit the growth of this microorganism. Similar trends were observed in similar publications [29,45,55]. For example, Martau et al. [29] analyzed the effect of fermentation with *Fructolactibacillus florum* DSM 22689 in single and in co-culture with *Saccharomyces cerevisiae* on wheat flour dough enriched with byproducts from apple juice production and obtained a final pH value of sourdoughs between 3.27 and 4.24. Paucean et al. [59] also demonstrated that through fermentation with single and co-cultures (*Lb. plantarum* ATCC 8014, *Lb. casei* 393, and *S. cerevisiae* ATCC 58523), by utilizing various substrates that contain carbohydrates such as glucose, fructose, sucrose, or maltose, the final pH value can be lowered significantly to values within the range of 3.5–4.5.

### 3.2. Organic Acids and Secondary Metabolites Production

In the fermentation with BB-12, the carbohydrates glucose, maltose, xylose, and xylo-oligosaccharides, together with the organic, lactic, and acetic acids, were considered. Based on the data obtained from the HPLC-RID, the glucose concentration decreased or was totally consumed until the end of fermentation (Table 1). The same trend was observed regarding XOS consumption.

Lactic and acetic acid have considerable importance in generating flavors in bakery products. Lactic acid at the fermentation outset was almost insignificant, with values between 0.01 and 0.12 mg/L. However, at the end of fermentation through glucose metabolization, it reached the highest value on the substrate, with 100% WF 1.98 ± 0.07 mg/L. There was a significant difference (*p* < 0.05) between this value and the values of batches with 5 (0.74 ± 0.06, 1.17 ± 0.09) and 10% (1.24 ± 0.10, 1.28 ± 0.11) of glucose or XOS addition, as evidenced in Table 1. With the help of starch hydrolysis, through substrate metabolism, glucose or XOS was steadily consummated by the bifidus pathway and generated lactic and/or acetic acid in the 48 h fermentation period. The accumulation of these organic acids also contributed to the acidification of the substrate, which decreased the pH and, after 24 h, began to inhibit microbial growth. The generation of these main metabolites can also contribute to validity extension, enhance antimicrobial activity, and upgrade the sensorial characteristics of bakery foodstuffs [60]. In comparison with heterofermentative bacteria in monoculture, the acetic acid content was insignificant in similar studies. However, with co-cultures (*F*. *sanfranciscensis* and *L. brevis*), content of 0.01 mmol/g [61], and with *F. florum* and *S. cerevisiae* co-culture, the highest value was obtained 0.30 ± 0.02 g/L on substrate enriched with 5% apple pomace [29]. Additionally, the formation of lactic acid was proved to be beneficial by improving the sensorial quality of gluten-free products [62].

Acetic acid production (Table 2) was observed as a small end product of batches with 100% WF, and there was no production observed on substrates where glucose was added. The acetic acid production increased with the increase in XOS addition, reaching a final value of 0.87 ± 0.03 mg/L, 0.59 ± 0.04, and 0.16 ± 0.02 in batches with 10, 5, and 2% XOS, respectively. As BB-12 uses the fructose-6-phosphate phosphoketolase pathway, it can generate more lactic and acetic acid than hetero- or homofermentative bacteria, and this can be the answer as to why, by XOS addition, these microorganisms produced acetic acid, whereas without any XOS, acetic acid was not found at all [34]. This aspect is also supported by the fact that the *Bifidobacterium* species use XOS as energy wellsprings and, as reported in human studies, the viability and the total bacterial count of this species increases in feces and decreases after the suspension of XOS supplementation [57]. Moreover, organic acid production has the outcome of moderate proteolysis through baking. Additionally, supplementation of doughs with organic acids increases the ductility of bakery products, as well as their finite volume and elasticity [63]. Paramithiotis et al. [60] analyzed the improved rheological characteristics of dough enriched with different organic acids, such as lactic, acetic, fumaric, malic, and citric acid. They proved that the integration or formation of organic acids through fermentation could enhance dough quality and diminish the final product’s hardness and sugar content.

The addition of XOS can be observed in Table 3, and with the consumption of XOS, the production of xylose can be observed, which can be explicated by the decomposition of XOS, which is constructed of 2 to 10 xylose units. The highest xylose production can be observed where 10% of XOS was added in a quantity of 1.62 ± 0.02 mg/L. As reported by Dysvik et al. [61], when sour beer was produced, the acetic acid production increased through substrate supplementation with XOS, which enhanced the sensorial quality and reduced the fermentation period of sour beer from 1–3 years to a period of 4 weeks. Xylose could also account for the sweetness of bakery products, in addition to improving the nutritional aspect of these foodstuffs. Moreover, XOS, besides being non-caloric, is effectively used in food production due to its high stability at high temperatures and low pH [64].

The substrate intensively influenced acetic and lactic acid formation in every batch. Although, regarding substrates with XOS addition, the glucose content was shallow and no significant lactic acid production could be observed, acetic acid production was quite elevated due to the enrichment of the substrate with XOS.

### 3.3. Rheological Analysis

**Figure 2:**
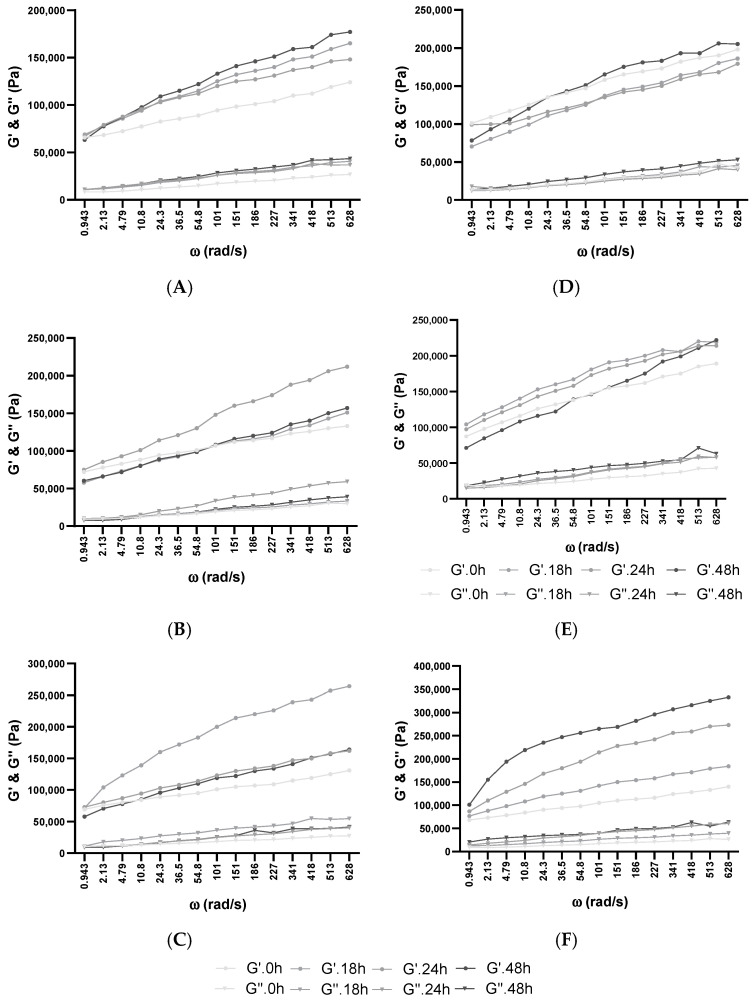
Influence of fermentation and frozen storage with BB-12 in fresh samples—(**A**) 100% WF, (**B**) 1% Glu, and (**C**) 1% XOS—and after frozen storage—(**D**) 100% WF, (**E**) 1% Glu, and (**F**) 1% XOS. Angular frequency (ω), storage modulus (G’), loss modulus (G”).

**Figure 3:**
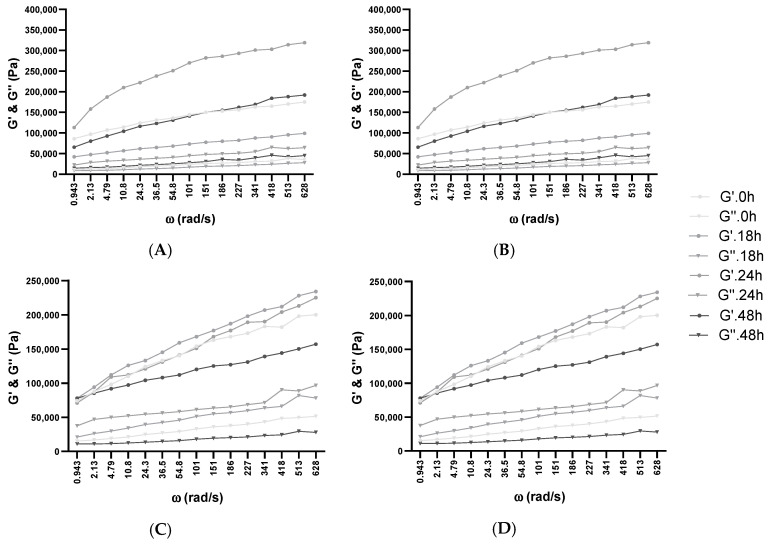
Influence of fermentation and frozen storage with BB-12 in fresh samples—(**A**) 5% Glu; (**B**) 5% XOS—and after frozen storage—(**C**) 5% Glu; (**D**) 5% XOS. Angular frequency (ω), storage modulus (G’), loss modulus (G”).

**Figure 4:**
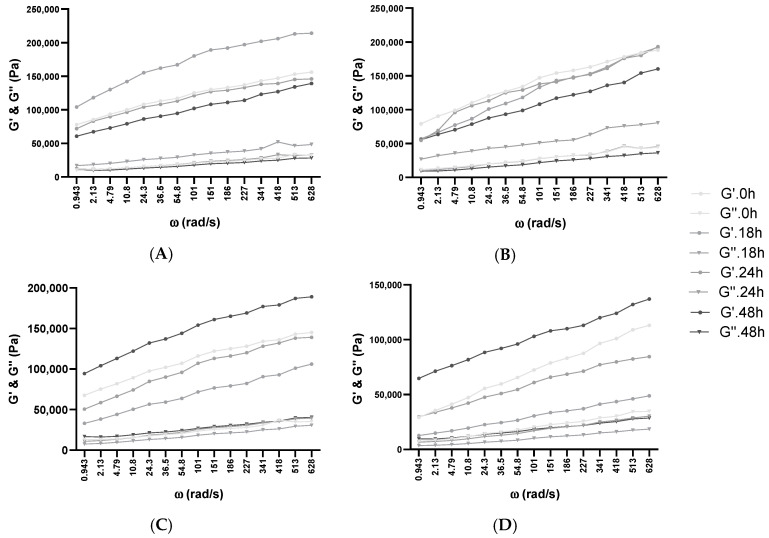
Influence of fermentation and frozen storage with BB-12 in fresh samples—(**A**) 10% Glu; (**B**) 10% XOS—and after frozen storage—(**C**) 10% Glu; (**D**) 10% XOS. Angular frequency (ω), storage modulus (G’), loss modulus (G”).

Viscoelastic solids (such as dough in this case) fermented with BB-12 were measured via dynamic rheological measurements. These tests may provide details concerning the dough behavior of the fermentation in each mixture. Each batch with or without XOS or glucose addition was measured previously and after 30 days of frozen storage. The prelevated samples were studied at 30 °C over 48 h (0, 18, 24, 48 h) of fermentation in a fresh state or after thawing at room temperature, as represented in Figure 2, Figure 3 and Figure 4 and Supplementary Figure S1. In each case, the increase in angular frequency (ω) from 0.628 to 628 rad/s revealed a growth in storage modulus (G’) and loss modulus (G”), which demonstrated a solid-like behavior. This increase also explains that dough recovery was slow following stress employment and happens as a consequence of an inadequately elastic network. The growth of G’ and G” could also be observed in our group’s previous studies, where WF enriched with soy flour was fermented with *Lb. plantarum*, *Lb. casei*, and *S. cerevisiae* in single and co-cultures [29,45,49], as well as other similar studies using different WF dough enriched with other flour types [54,65].

Water dispersion and protein polymerization play a fundamental role in the supervision of dough behavior throughout hydration and growth, which confirms the characteristic of the end product [54]. These two aspects are strongly related to the conformational stability of the proteins and the dough’s viscoelasticity [66]. Additionally, several studies indicate that WF dough and its corresponding gluten (WF moisturized proteins) exhibit viscoelastic behavior. Gluten can be distinguished by its bimodal division among the polymeric glutenin and monomeric gliadin proteins [67]. Because the degree of humidity influences the dynamic moduli [67] and the absorption capacity of XOS, glucose, and WF are different, the same amount of water was added prior to inoculation in the case of every dough mixture. The results indicated that sample viscosity did not increase significantly with the addition of XOS or glucose. Still, increased viscosity could be observed from the beginning of fermentation until the end through every fermentation. Based on recent studies on cookie dough, it was proven that XOS supplementation modifies the water-retention and binding capability of dough [39]. The used high-purity XOSs are considered for food industry applicability in particular, and they consist of 70–95% XOS. This study also reported that supplementation with XOS in aqueous gels, in comparison with sucrose or FOS, increased the viscosity at room temperature, although at higher temperatures, no significant modification in viscosity could be observed.

The loss factor (tan δ) offers details regarding the molecular synergies of the materials, where completely organized materials have a decreased tan δ value [68]. The growing trend corresponding to the frequency amplification across all analyzed dough mixtures was acquired, demonstrating their viscous behavior (Supplementary Figure S2). A decline in elastic behavior was observed at increased frequencies. Additionally, with the increase in XOS and glucose in WF dough, the elastic behavior was additionally amplified. This can be attributed to the enhancement of the polymer quantity or the presence of more water-dissolvable solids in batches with 10% XOS and 10% glucose addition, resulting in behavior being more elastic than viscous. The expansion of tan δ values was comparable with the frequency growth in every batch. Similar behavior has been described through the incorporation of dairy elements by substituting water with acid whey or milk in WF doughs [69], or in the case of whey protein isolate enrichment of WF and rice flour doughs by analyzing, in rice crackers, the suitableness of the protein-hydrocolloid composite substitution in favor of WF proteins [70]. Consequently, in every examined frequency sweep, G’ was superior to G” in fresh and thawed samples, which manifested in preeminence a more elastic solid-like characteristic, as evidenced by the loss of tangent values situated under 1. The highest moduli values were obtained in the 10% XOS sample after frozen storage, which showed a lower elastic property and higher viscosity. This aspect is in concordance with previous studies, where the addition of small amounts of XOS (1–3 *w*/*w*%) did not influence the rheological properties of foodstuffs, whereas in higher quantities, it has a texture-modifying potential [39].

As bakery products are predisposed to drying, along with a decline in quality and increased tastelessness, a par-baking procedure can be applied, which was elaborated for the first time in the 1980s, making available fresher and tastier commodity materials [70]. However, frozen storage and increased freezing duration is reported to cause a decline in the quality of the final product. This is why rheological characteristic assessment is implemented to evaluate the consequences of dough structure, as it holds an essential connection with the interplay among components. Many flours (soybean, quinoa, durum, amaranth, etc.) and hydrocolloids can enhance flour stability during frozen storage [30,71,72]. These flours, as well as XOS supplementation, can be effectively implemented to ameliorate doughs, particularly their viscous texture [73].

Organic acid production (lactic and acetic) and carbohydrate metabolization had a significant effect on the viscoelastic property of doughs. For example, in the case of 10% glucose and XOS addition, the former had lower viscoelasticity than the latter, which can be attributed to the fact that lactic and acetic acid was also produced (1.28 ± 0.11, 0.87 ± 0.03). These metabolites acted as acidifying agents, and with the decrease in pH, a change occurred in the mechanical properties.

## 4. Conclusions

The physicochemical features of fresh and frozen dough enriched with XOS or glucose presented significant physicochemical divergences. Through aerobic fermentation with the probiotic strain BB-12, the carbohydrates, namely glucose, maltose, XOS, and xylose, were substantially consumed, and lactic and acetic acid was produced. The production of acetic acid was only observed on substrates enriched with XOS. The highest acetic acid quantity of 0.87 ± 0.03 mg/L was obtained in 10% XOS, and in the same substrate, the final lactic acid was produced in an amount of 1.28 ± 0.11 mg/L. The high quantity of organic acids also positively influenced the rheological properties of dough by improving elasticity behavior before and after frozen storage. However, dough enrichment with XOS or glucose did not significantly improve the viability of BB-12.

The limitations of this investigation include the accumulation of the generated metabolites and organic acids, oxygen conditions, the utilization of single culture, and the absence of the final product. To overcome these limitations, further studies under aerobic and anaerobic conditions could be investigated for the effects of XOS supplementation on the stability of food products, physicochemical parameters, and organoleptic properties. Furthermore, another practical application would be the utilization of co-cultures for a better fermentation of these oligosaccharides.

## Figures and Tables

**Table 1:** Quantity of glucose, maltose, and lactic acid (mg/L) during fermentation.

	Time (h)	100% WF	1% Glu	2% Glu	5% Glu	10% Glu	1% XOS	2% XOS	5% XOS	10% XOS
Glucose	0	2.04 ± 0.14 ^c^	2.93 ± 0.14 ^c^	7.25 ± 0.22 ^bc^	11.38 ± 0.16 ^b^	29.16 ± 0.32 ^a^	1.24 ± 0.08 ^cd^	0.31 ± 0.04 ^d^	0.32 ± 0.06 ^d^	0.15 ± 0.03 ^d^
	18	0.59 ± 0.06 ^d^	2.61 ± 0.13 ^c^	4.05 ± 0.19 ^b^	11.26 ± 0.25 ^b^	42.36 ± 0.29 ^a^	0.33 ± 0.05 ^d^	0.21 ± 0.02 ^d^	0.19 ± 0.02 ^d^	0.06 ± 0.01 ^d^
	24	0.20 ± 0.07 ^d^	1.66 ± 0.09 ^c^	4.03 ± 0.16 ^b,c^	12.03 ± 0.24 ^b^	41.63 ± 0.36 ^a^	0.21 ± 0.03 ^d^	0.21 ± 0.02 ^d^	0.16 ± 0.02 ^d^	0.09 ± 0.01 ^d^
	48	0.63 ± 0.09 ^e^	1.84 ± 0.09 ^d^	4.36 ± 0.14 ^c^	9.59 ± 0.14 ^b^	33.20 ± 0.35 ^a^	0.17 ± 0.02 ^e^	0.17 ± 0.01 ^e^	N.D.	N.D.
Maltose	0	0.41 ± 0.04 ^d^	5.69 ± 0.15 ^a,b^	3.59 ± 0.21 ^b^	2.14 ± 0.16 ^b,c^	1.32 ± 0.10 ^c^	5.13 ± 0.12 ^a,b^	2.18 ± 0.09 ^b,c^	1.21 ± 0.10 ^b^	6.59 ± 0.24 ^a^
	18	10.11 ± 0.11 ^a^	4.41 ± 0.12 ^c,d^	4.58 ± 0.19 ^c,d^	2.64 ± 0.12 ^d,e^	1.86 ± 0.13 ^e^	7.75 ± 0.14 ^b^	1.93 ± 0.11 ^c^	1.35 ± 0.09 ^e^	2.23 ± 0.12 ^d,e^
	24	6.71 ± 0.23 ^a,b^	2.11 ± 0.09 ^c^	4.83 ± 0.14 ^b^	2.89 ± 0.10 ^c^	1.85 ± 0.09 ^c^	7.97 ± 0.21 ^a^	1.27 ± 0.08 ^c^	0.92 ± 0.05 ^c^	2.39 ± 0.10 ^c^
	48	10.37 ± 0.19 ^a^	0.45 ± 0.06 ^d^	6.01 ± 0.18 ^b^	2.41 ± 0.10 ^c^	1.09 ± 0.06 ^d^	9.72 ± 0.10 ^a^	0.87 ± 0.05 ^d^	0.67 ± 0.04 ^d^	0.18 ± 0.08 ^d^
Lactic acid	0	0.01 ± 0.00 ^a^	0.06 ± 0.00 ^a^	0.06 ± 0.01 ^a^	0.09 ± 0.01 ^a^	0.03 ± 0.00 ^a^	0.02 ± 0.00 ^a^	0.12 ± 0.01 ^a^	0.01 ± 0.00 ^a^	0.09 ± 0.01 ^a^
	18	0.53 ± 0.02 ^a,b^	0.83 ± 0.06 ^a^	0.43 ± 0.05 ^b^	0.37 ± 0.03 ^b^	0.51 ± 0.04	0.76 ± 0.04 ^a^	0.24 ± 0.01 ^b^	0.56 ± 0.04 ^a,b^	1.07 ± 0.09 ^a^
	24	0.74 ± 0.03 ^b^	0.93 ± 0.08 ^a,b^	0.52 ± 0.05 ^b^	0.51 ± 0.05 ^b^	0.66 ± 0.05 ^b^	1.08 ± 0.10	0.23 ± 0.02 ^c^	0.90 ± 0.07 ^a,b^	1.44 ± 0.11 ^a^
	48	1.98 ± 0.07 ^a^	0.95 ± 0.10 ^b^	1.22 ± 0.08 ^a,b^	0.74 ± 0.06 ^b,c^	1.24 ± 0.10 ^a,b^	1.50 ± 0.10 ^a^	0.30 ± 0.03 ^c^	1.17 ± 0.09 ^a,b^	1.28 ± 0.11 ^a,b^

Data displayed as mean of triplicates ± SD. In every row, significant differences (*p* < 0.05) are displayed with different letters (a–e) between the variety of substrate used (one-way ANOVA, multiple comparisons test, together with Tukey multiple range test (*p* = 0.05). WF—wheat flour, XOS—xylo-oligosaccharides, Glu—glucose.

**Table 2:** Acetic acid (mg/L) quantity during fermentation.

	Time (h)	100% WF	1% XOS	2% XOS	5% XOS	10% XOS
Acetic acid	0	N.D.	N.D.	N.D.	N.D.	0.16 ± 0.01
	18	N.D.	N.D.	0.11 ± 0.01 ^b^	0.26 ± 0.02 ^b^	0.77 ± 0.07 ^a^
	24	N.D.	0.12 ± 0.01 ^b^	0.12 ± 0.01 ^b^	0.40 ± 0.03 ^b^	0.95 ± 0.08 ^a^
	48	0.19 ± 0.01 ^b^	0.22 ± 0.02 ^b^	0.16 ± 0.02 ^b^	0.59 ± 0.04 ^a,b^	0.87 ± 0.03 ^a^

Data displayed as mean of triplicates ± SD. In every row, significant differences (*p* < 0.05) are displayed with different letters (a,b) between the variety of substrate used (one-way ANOVA, multiple comparisons test, together with Tukey multiple range test (*p* = 0.05). WF—wheat flour, XOS—xylo-oligosaccharides, Glu—glucose.

**Table 3:** Quantity of xylose and XOS (mg/L) during fermentation.

	Time (h)	1% XOS	2% XOS	5% XOS	10% XOS
XOS	0	0.04 ± 0.00 ^d^	2.78 ± 0.08 ^c^	5.83 ± 0.09 ^b^	13.08 ± 0.15 ^a^
	18	N.D.	2.92 ± 0.06 ^c^	5.11 ± 0.11 ^b,c^	17.29 ± 0.18 ^a^
	24	N.D.	2.44 ± 0.09 ^c^	4.24 ± 0.08 ^b^	14.43 ± 0.12 ^a^
	48	N.D.	2.72 ± 0.04 ^b^	2.67 ± 0.06 ^b^	5.93 ± 0.11 ^a^
Xylose	0	0.75 ± 0.04 ^a^	0.52 ± 0.04 ^a^	0.40 ± 0.02 ^a^	0.746 ± 0.06 ^a^
	18	0.58 ± 0.02 ^b^	0.28 ± 0.02 ^b^	0.45 ± 0.02 ^b^	1.207 ± 0.06 ^a^
	24	0.78 ± 0.04 ^a,b^	0.25 ± 0.02 ^c^	0.64 ± 0.03 ^b^	1.20 ± 0.02 ^a^
	48	1.12 ± 0.06 ^a,b^	0.29 ± 0.02 ^b^	1.03 ± 0.04 ^a,b^	1.62 ± 0.02 ^a^

Data displayed as mean of triplicates ± SD. In every row, significant differences (*p* < 0.05) are displayed with different letters (a–d) between the variety of substrate used (one-way ANOVA, multiple comparisons test, together with Tukey multiple range test (*p* = 0.05). XOS—xylo-oligosaccharides.

## Data Availability

Not applicable.

## Supplementary Materials

The following supporting information can be downloaded at: https://www.mdpi.com/article/10.3390/biology11040553/s1. Figure S1: Influence of fermentation and frozen storage with BB-12 in fresh samples A. 2% Glu; B. 2% XOS; and after frozen storage C. 2% Glu; D. 2% XOS; angular frequency (ω), storage modulus (G’), loss modulus (G”); Figure S2: Influence of fermentation and frozen storage with BB-12 in fresh samples and after frozen storage A. 100% WF; B. 1% Glu, C. 1% XOS, D. 2% Glu, E. 2% XOS, F. 5% Glu, G. 5% XOS, H. 10% Glu, I. 10% XOS.

## Disclaimer

Gabriela Precup is currently employed as Scientific Assistant since April 2021 by Randstad (c/o EFSA) in the Nutrition and Food Innovation Unit that provides scientific and administrative support to the Panel on Nutrition, Novel Foods and Food Allergens in the area “Safety Assessment of Novel Foods”. She was also employed as a trainee at the European Food Safety Authority (EFSA) between 1st November 2019 and 30th April 2021 in the Nutrition Unit. However, the present article is published under the sole responsibility of the authors Gabriela Precup, Bernadette-Emőke Teleky, Floricuța Ranga, and Dan Cristian Vodnar and may not be considered as an EFSA scientific output. The positions and opinions presented in this article are those of the author/s alone and are not intended to represent the views/any official position or scientific works of EFSA. To learn more about the views or scientific outputs of EFSA, please consult its website at http://efsa.europa.eu (accessed on 16 December 2021).
