# Supplementary material for: Assessment of Physicochemical and Rheological Properties of Xylo-Oligosaccharides and Glucose-Enriched Doughs Fermented with BB-12
Source: Biology (Basel). 2022 Apr 2;11(4):553. doi: 10.3390/biology11040553 (PMC9027653; doi:10.3390/biology11040553)
Supplement: Supplementary file 1 [file biology-11-00553-s001.zip › biology-1637816-supplementary.pdf]

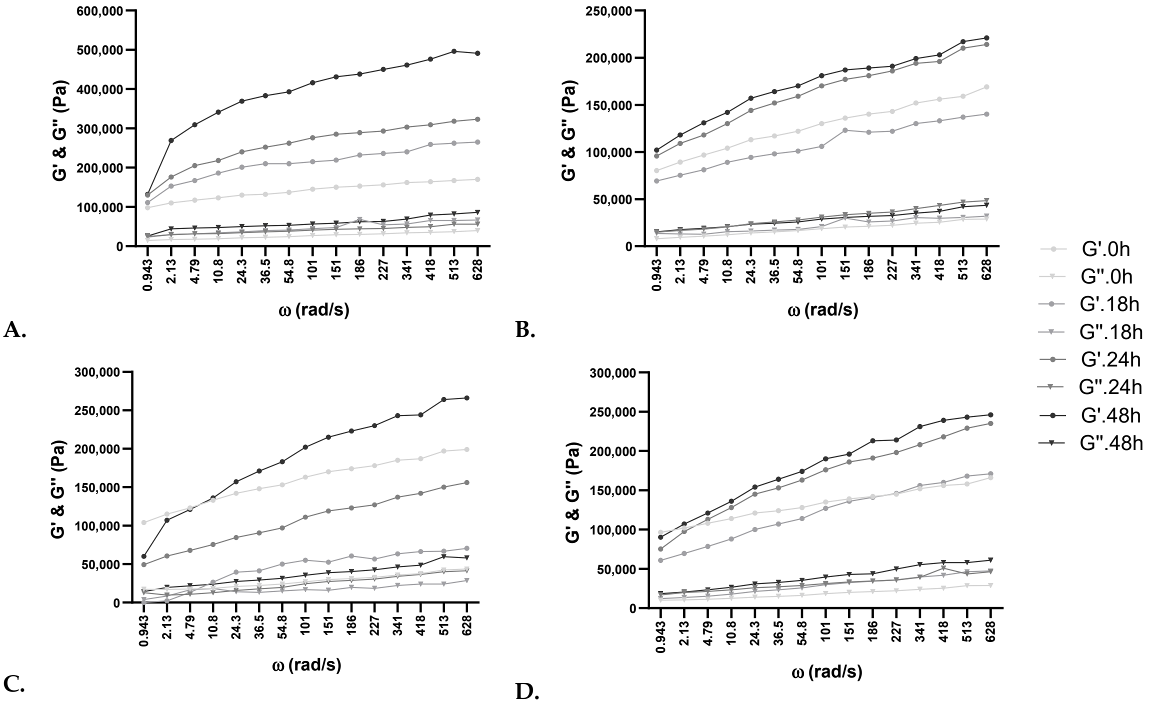

Supplementary Figure S1. Influence of fermentation and frozen storage with BB-12 in fresh samples A. 2% Glu; B. 2% XOS; and after frozen storage C. 2% Glu; D. 2% XOS; angular frequency (ω), storage modulus (G'), loss modulus (G'').

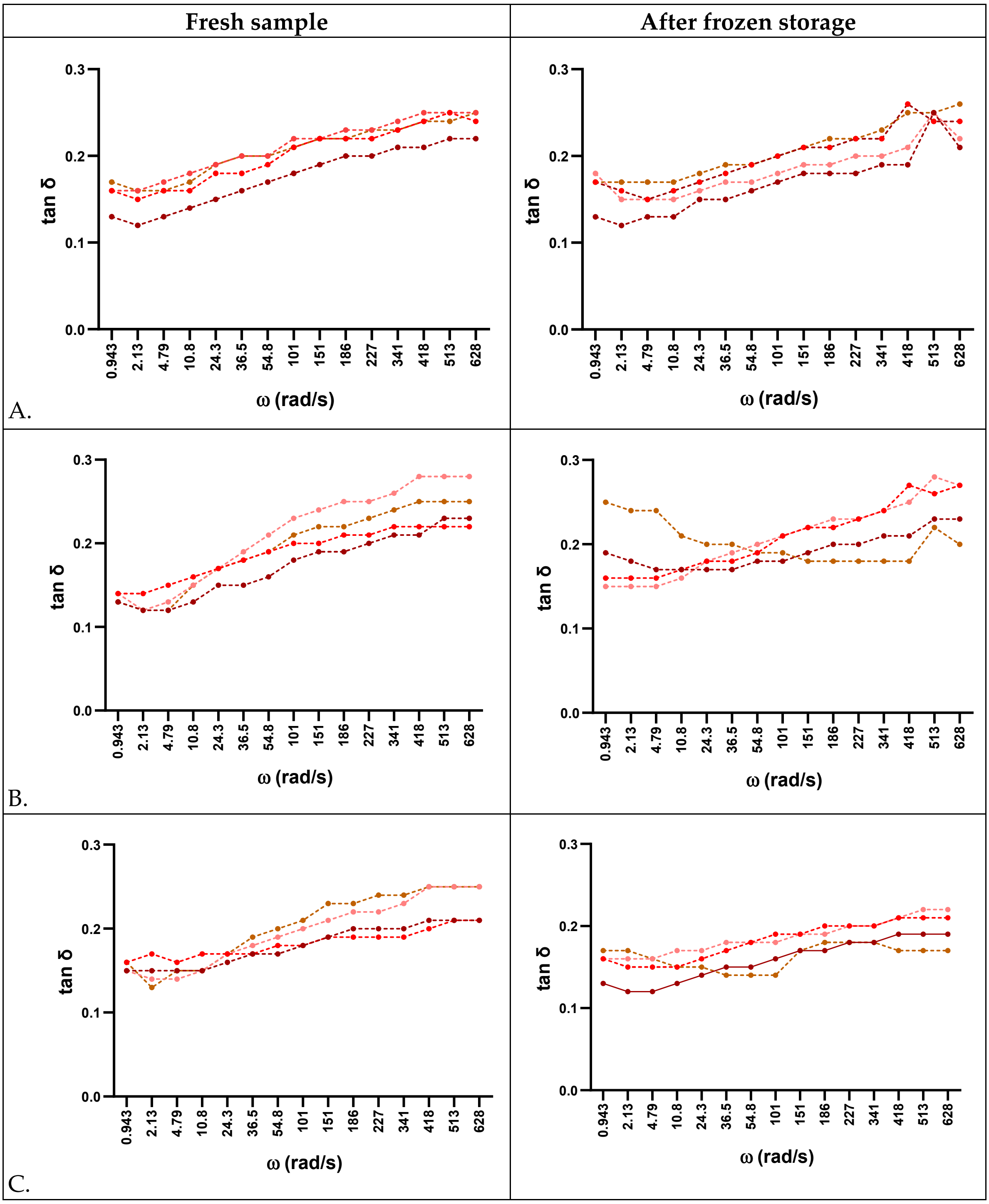
Fresh sample
After frozen storage
tan δ
ω (rad/s)
0.0
0.1
0.2
0.3
0.943
2.13
4.79
10.8
24.3
36.5
54.8
101
151
186
227
341
418
513
628
A.
B.
C.

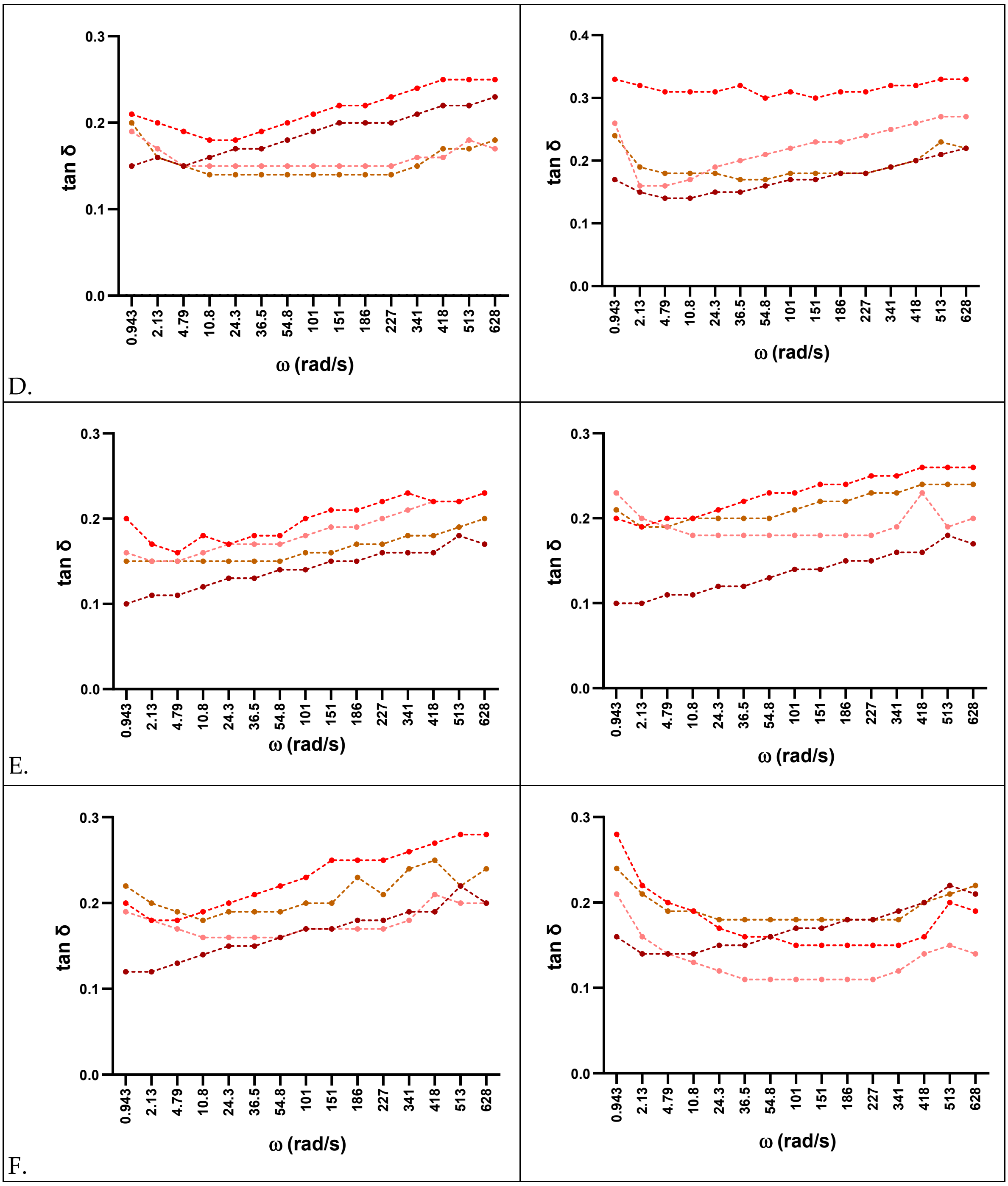
D.
E.
F.
tan δ
ω (rad/s)
0.0
0.1
0.2
0.3
0.4
0.943
2.13
4.79
10.8
24.3
36.5
54.8
101
151
186
227
341
418
513
628

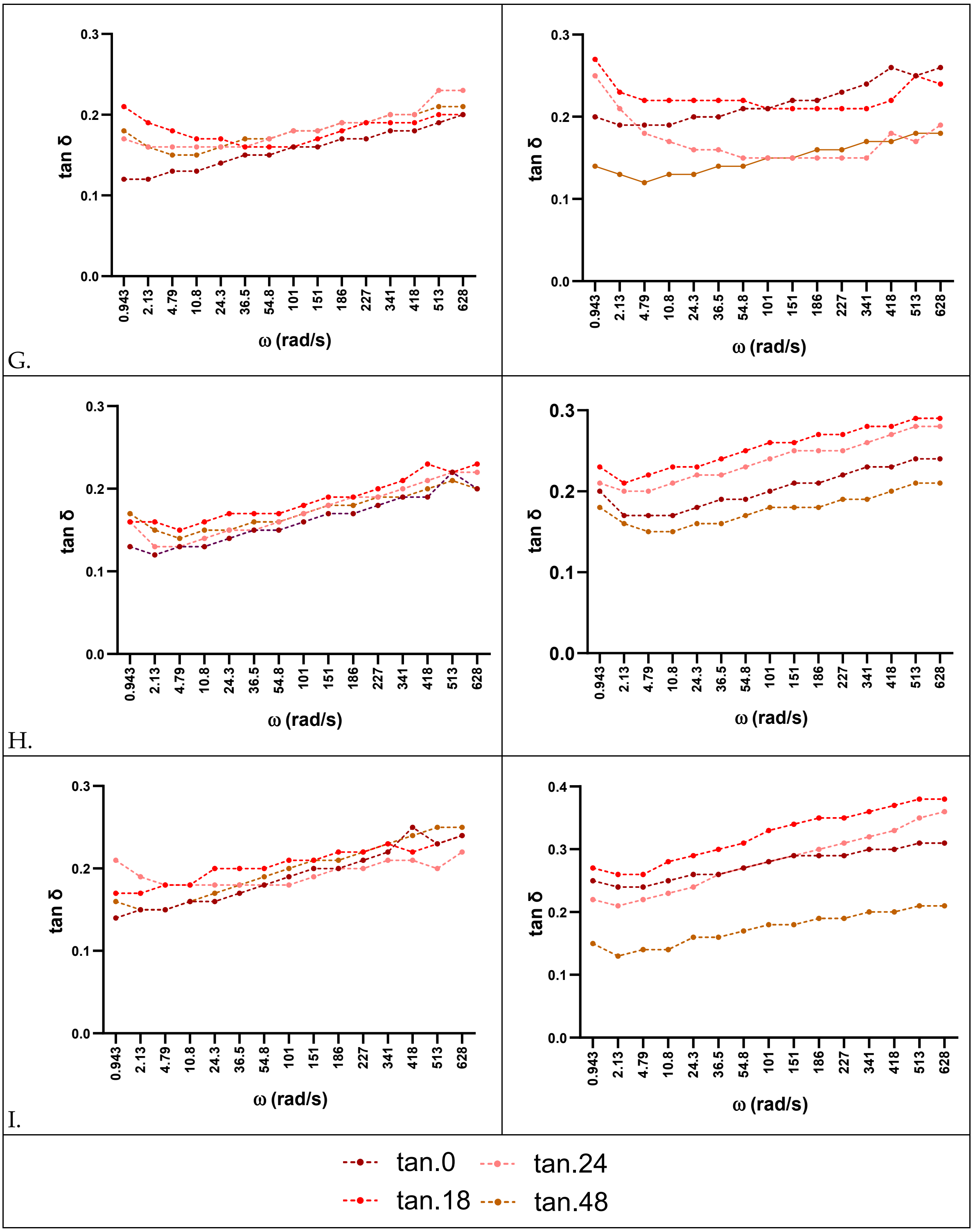

Supplementary Figure S2. Influence of fermentation and frozen storage with BB-12 in fresh samples and after frozen storage A. 100% WF; B. 1% Glu, C. 1% XOS, D. 2% Glu, E. 2% XOS, F. 5% Glu, G. 5% XOS, H. 10% Glu, I. 10% XOS.angular frequency (ω), storage modulus (G'), loss modulus (G'')
